# Investigating the Effect of Dexmedetomidine in Controlling Postoperative Emergence Agitation in Children under Sevoflurane Anesthesia

**DOI:** 10.1155/2024/6418429

**Published:** 2024-07-24

**Authors:** Mitra Golmohammadi, Shahryar Sane, Somayeh Ghavipanjeh Rezaei, Rana Hosseini, Enas R. Alwaily, Beneen M. Hussien, Ramin Mohammadpour, Nazila Rahmani, Behzad Kazemi Haki

**Affiliations:** ^1^ Department of Anesthesiology Urmia University of Medical Sciences, Urmia, Iran; ^2^ Department of Nursing School of Nursing and Midwifery Maragheh University of Medical Sciences, Maragheh, Iran; ^3^ Department of Social Medicine Urmia University of Medical Sciences, Urmia, Iran; ^4^ Microbiology Research Group College of Pharmacy Al-Ayen University, Thi-Qar, Nasiriyah, Iraq; ^5^ Medical Laboratory Technology Department College of Medical Technology The Islamic University, Najaf, Iraq; ^6^ College of Medical Veterinary and Life Science University of Glasgow, Glasgow, UK

## Abstract

**Introduction:**

Emergence agitation (EA) is one of the common problems during recovery from general anesthesia, especially in children. In this study, we investigated the effect of dexmedetomidine on the control of agitation after anesthesia with sevoflurane in children.

**Method:**

This randomized control-placebo, double-blind prospective clinical trial was conducted on seventy-six children between 2 and 7 years with ASA (American Society of Anesthesiologists) class I who were candidates for elective adenoidectomy surgery and tonsillectomy. Participants were selected by an available sampling method. Patients were randomly placed in one of the two groups D (dexmedetomidine 0.5 *μ*g/kg infusion within ten minutes) or P (placebo: normal saline infusion within ten minutes). A four-point scale evaluated agitation. Pain evaluation was done by FLACC (faces, legs, activity, cry, and consolability). The statistical software was SPSS version 23. *P* < 0.05 was considered statistically significant.

**Results:**

The level of agitation was significantly lower in the intervention group (*P* < 0.05), except after 40 minutes in the PACU (Post Anesthesia Care Unit) (*P*=1.00). Patients in the control group experienced high pain scores when admitted at PACU, 10, 20, and 30 minutes after admission at PACU (*P* < 0.05). Pethidine and metoclopramide prescriptions in the intervention group were lower than in the control group (*P* < 0.05). Shivering occurred in five patients in the intervention group and nine in the control groups (*P*=0.032). Hypotension that required intervention occurred in 3 patients in the intervention group and one in the control group (*P*=0.024).

**Conclusion:**

Our trial demonstrated that the prescription of 0.5 *μ*g/kg of dexmedetomidine within ten minutes after intubation significantly reduced the EA frequency, pain severity, analgesic consumption, and PONV (postoperative nausea and vomiting). However, it caused delays in the emergence from anesthesia. This trial is registered with IRCT20160430027677N14.

## 1. Introduction

Agitation after awakening was first expressed in 1960 and was defined as a dissociated phase of awakening that is accompanied by a series of emotional reactions so that the child becomes restless and even inconsolable. Children have no cooperation and sometimes show aggressive behavior [1, 2]. This complication is very stressful for children, their parents, and the medical staff. Despite the relatively large number of articles on EA (emergence agitation), there is limited information about its prevalence, pathophysiology, causes, or even its control or treatment. EA prevalence in the papers, depending on the determining method and criteria used by researchers, is reported between 25% and 80% [3]. It seems that a series of possible causes are effective in its occurrences, such as preschool age, anxiety before surgery, personality characteristics, the early awakening after anesthesia, presence of pain, kind of surgery (such as eye or ear, nose, and throat surgeries), technique and type of drugs prescribed for anesthesia (Newer Inhalation Anesthesia agents such as isoflurane, sevoflurane, and desflurane), and even environmental issues [2–4]. EA can have clinically significant consequences, such as the patient's self-injury, which causes pain or may even cause bleeding from the surgical site, difficulty in taking care of a restless child, prolonged recovery time, the need for additional medical care, and finally, increased costs [4–6].

Sevoflurane is a halogenated inhalational anesthetic agent used to induce and maintain general anesthesia and widely used in pediatric anesthesia due to its lack of unpleasant odor, low blood-to-gas solubility ratio, rapid anesthesia induction, the early awakening of patients, stable hemodynamic maintenance, mild cardiovascular system depression, and low liver toxicity. Although there are many advantages to anesthesia with sevoflurane, agitation when coming out of anesthesia (emergence agitation “EA”) is one of the common side effects of anesthesia with this drug in children [7].

Various drugs such as opiates, benzodiazepines, ketamine, propofol, sodium thiopental, and *α*2-agonists such as dexmedetomidine have been used to prevent and treat this condition. Still, the results regarding their effectiveness are different and are always discussed [8]. Meanwhile, a meta-analysis research found evidence in favor of dexmedetomidine being used as a premedication to control agitation in children undergoing both painful and nonpainful procedures [9]. Nonetheless, it suggests that more research be done to determine the ideal DEX dosage, route, patterns, and timing as well as how it affects other results. Therefore, it appears that it will also be helpful in reducing children's agitation following surgery.

Dexmedetomidine is a specific *α*2 adrenoceptor agonist with a ratio (*α*2/*α*1 = 1620/1). In addition, the drug has sedative and pain-relieving properties [10]. The sedative effect is caused by its action on *α*2 presynaptic receptors in the locus coeruleus, which reduces the release of adrenaline and facilitates the inhibitory activity of neurons, especially the gamma-aminobutyric acid system. Its analgesic effect is created through the *α*2 receptor on the posterior horn of the spinal cord and the supraspinal cord, reducing the secretion of substance P [11]. It should be noted that although the sedative dose of dexmedetomidine does not cause respiratory depression, it can cause cardiovascular effects, so it needs careful monitoring.

Some studies have indicated that dexmedetomidine can reduce the prevalence of EA caused by sevoflurane in pediatric patients. However, it is controversial, and they announced that more studies are required to evaluate the effect of dexmedetomidine on the prevention of PONV [9, 12]. Therefore, according to the mentioned points and the lack of such a study in our society, this clinical trial investigated the effect of dexmedetomidine in preventing or reducing the prevalence of agitation after anesthesia when coming out of anesthesia in children under general anesthesia with sevoflurane.

## 2. Methods

In this randomized control-placebo, double-blind prospective clinical trial, after acquiring ethical approval from the university's ethics committee (ID: IR.UMSU.REC.1397.411 and Date: 2019-01-16), parents were sufficiently explained by the anesthesiologist about the procedure and performing the trial, and then parents signed the consent form. Seventy-six children between 2 and 7 years with ASA class I who were candidates for elective adenoidectomy surgery, tonsillectomy, or both were selected by an easy and available sampling method. The study started on 2019-05-11, and sampling ended on 2019-07-26. After selecting one of 76 cards (containing cover) by parents, patients were randomly placed in one of the two groups D (dexmedetomidine) or P (placebo), where each group consisted of 38 people. This double-blind, randomized clinical trial is registered in Iranian Randomized Clinical Trials (ID: IRCT20160430027677N14 and Date: 2019-05-07).

### 2.1. Sample Size

Based on the mean FLACC and Ramsy sedation scores in the Sadeghi et al. study, which compared the efficacy of early versus late administration of dexmedetomidine on EA in children undergoing oral surgery [13], and according to the formula for determining the sample size of analytical studies to compare the estimation of proportions in two groups (Pocock's method) and considering the test power of 90% and 95% confidence interval, the required sample size (considering the possibility of a 10% sample drop) for each group was determined to be 38 people.

(1) $$\begin{array}{c} N=\frac{P1\left(100-P1\right)+P2\left(100-P2\right)}{\left(P2-P1\right)}. \end{array}$$

### 2.2. Inclusion Criteria

The inclusion criteria were patients (male and female) aged 2 to 7, ASA classes I (American Society of Anesthesiology), and candidates for elective adenoidectomy surgery, tonsillectomy, or both.

### 2.3. Exclusion Criteria

Exclusion criteria were intellectual or developmental disability (IDD) or presence of neurological diseases; upper airway abnormality; children with comorbid cardiovascular systemic diseases or ongoing allergies to the study drug; history of asthma or other lung diseases; and presence of lung infections in the last four weeks. Moreover, children with special conditions during the operation such as severe hypotension, malignant arrhythmia, significantly prolonged operation time for other reasons, parents who refused to participate in the study, and children who consumed chronic painkillers were excluded.

### 2.4. Trial Design

Intervention group: the patients in the intervention group received an infusion of 0.5 *μ*g/kg/h of dexmedetomidine within ten minutes after induction of anesthesia.

Control group: the patients in the control group received an equal volume of normal saline infusion within ten minutes.

Six hours before the surgery, patients did not receive any solid food or milk; if necessary, they only consumed filtered liquids up to 2 hours before the surgery. None of the patients received premedication, and once patients arrived in the operating room, they were evaluated and scored in terms of agitation using the four-point scale method. Then they were subjected to routine monitoring such as electrocardiography, pulse oximetry, and noninvasive sphygmomanometer. Without the presence of the parents, the children were subjected to induction of anesthesia by preoxygenation with O2 100%, sevoflurane with its gradual increase up to a maximum of 8% and 100% oxygen. After losing consciousness, an intravenous line was inserted, 0.5 mg/kg of atracurium was injected, and then intubation was performed with an appropriate tracheal tube. Then, for each of the patients in the intervention group, 0.5 *μ*g/kg of dexmedetomidine, and in the placebo group, an equal volume of normal saline was infused within ten minutes. It should be noted that dexmedetomidine was taken from 100 micrograms per milliliter and diluted in a 50 ml syringe with normal saline to achieve a final 4 mcg/ml concentration. The desired dose was placed in a fifty-milliliter syringe and injected through a syringe pump. Of course, the same volume and method were also prepared and performed for the placebo group so that a 50 ml syringe containing normal saline was loaded through a syringe pump.

The method of preparing and injecting the drugs was done by an anesthetist who was unaware of which patient belonged to which group. The drug containers were similar. After data collection, the anesthesiologist and ENT specialist were informed about the patients' groups.

**Figure 1:**
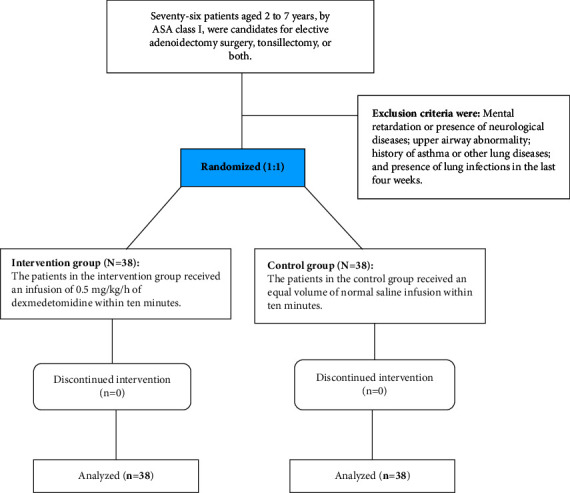
Study flow diagram.

In both groups, simultaneously with the injection of the study drugs, acetaminophen suppositories of 12 mg/kilogram and ondansetron 0.15 mg/kilogram were prescribed for each patient. Anesthesia was maintained with MAC (Minimum Alveolar Concentration) of 2–4% sevoflurane and 50% oxygen, and 50% nitrous oxide. During anesthesia, heart rate, mean arterial pressure, arterial oxygen saturation (SPO2), and end-tidal CO2 (EtCO2) were continuously monitored. A decrease in heart rate or blood pressure up to 10% of the baseline value was acceptable, and less than that was corrected by reducing anesthetic drugs and administering fluids. If it was ≤60 HR, atropine 0.02 microgram/kilogram was injected. At the end of the surgery, the anesthetic gases were turned off. The neuromuscular block was reversed by injecting 40 *μ*g/kg of neostigmine and 20 *μ*g/kg of atropine. After ensuring patients could maintain the gag reflex, the presence of mimicry on the face, and observing the limbs' movements in a targeted way, the tracheal tube was removed after oropharyngeal suction. The time between insertion and removal of the oral and pharyngeal pack was considered the surgery time, and the time from induction with sevoflurane to removing the endotracheal tube was considered the anesthesia time. The extubation time was calculated from the time of completion of the surgery to the removal of the endotracheal tube. The emergence period from anesthesia was defined as when the patient spontaneously opened their eyes for more than 5 seconds. When entering PACU, the heart rate, mean arterial pressure, arterial oxygen saturation (SPO2), and breathing rate were recorded continuously for 30 minutes by the anesthesia nurse in PACU, who was unaware of the grouping of patients. For all patients, we placed a hot air blower in the PACU unit. Parents were not allowed in PACU according to hospital internal policies. The study flow diagram is showed in Figure 1.

### 2.5. Randomization and Blinding

The participants and their parents were unaware of the groups' assignments. The randomization process was done using a random number generator to ensure the allocation was truly random. In this clinical trial, participants were randomly assigned to either the interventional or placebo according to the random number table (38 people in the intervention group and 38 in the control group), and then the study began.

### 2.6. Measurements

Evaluation of agitation after anesthesia was done at six-time intervals. The first time was after extubation, then the time of leaving the operating room, and then 10, 20, 30, and 40 minutes after the patient was in PACU, which was evaluated by an Aonos four-point scale [14] and included 1 = calm, 2 = not calm, but could be easily calmed, 3 = moderately agitated or restless, and 4 = combative, excited, disoriented. Scores of one and two were defined as the absence of EA after anesthesia, and scores of three and four were interpreted as the presence of emergence agitation after anesthesia. Pain evaluation was done by FLACC (faces, legs, activity, cry, and consolability) [15]. The FLACC scale assesses pain for children between 2 months and seven years or individuals unable to express their pain. The score 0 = relaxed and comfortable, 1–3 = mild discomfort, 4–6 = moderate pain, and 7–10 = severe discomfort/pain [15]. If EA ≥3 or FLACC ≥5, pethidine was injected at 0.5 mg/kg, and if the symptoms of agitation after anesthesia did not decrease or disappear, the same dose was repeated 20 minutes later. In case of vomiting, 0.15 mg/kg of metoclopramide was injected. In addition to the frequency of agitation after anesthesia, the duration of surgery and anesthesia, the time interval of shutting off the anesthetic gases and removing the tracheal tube, the length of PACU, and complications such as bradycardia, decrease in saturation <95% SPO2, vomiting, shivering, and laryngospasm were recorded. Finally, the patients were discharged from the PACU ward according to Aldrette's score [7].

### 2.7. Statistical Analysis

Data were reported using descriptive statistics (frequency and percentage) and mean ± standard deviation (mean ± SD). *T*-test was used to analyze quantitative data, and the chi-square test was used for qualitative variables. The statistical software used was SPSS version 23. *P* < 0.05 was considered statistically significant.

## 3. Results

The pediatrics' demographic data in the intervention and control groups are illustrated in Table 1. The chi-square and *t*-tests showed no statistically significant difference between the two groups' data regarding gender, age, BMI, duration of anesthesia, and surgery (*P* > 0.05). It demonstrated that both groups were homogeneous in terms of demographic characteristics. The mean time of emergence from anesthesia was significantly lower in the control group than in the intervention group (*P*=0.001).

### 3.1. Mean Heart Rate

The mean heart rate in the intervention group after the injection of dexmedetomidine was significantly lower than the control group. However, there was no significant difference between the heart rate at the other designated times (Table 2).

### 3.2. Mean Arterial Pressure

The mean arterial pressure at 5 and 10 minutes after starting the surgery was lower in the intervention group than in the control group, and it was statistically significant (*P* < 0.05). Moreover, there were significant differences between the two groups at times before transferring to PACU, when arriving at the PACU, and in the first 10 minutes of PACU (*P* < 0.05) (Table 3).

### 3.3. Incidence of Agitation

The incidence of emergence agitation after anesthesia in the control group was significantly higher than in the intervention group (*P* < 0.05). So, 53.95% of patients in the control group and 34.21% in the intervention group had emergence agitation after anesthesia (Table 4).

The mean stay in PACU was 40 ± 6.27 minutes in the control group and 42.32 ± 5.73 minutes in the intervention group. There was no significant difference between the length of stay in PACU between the two study groups (*P*=0.183).

### 3.4. The Mean Pain Score

**Figure 2:**
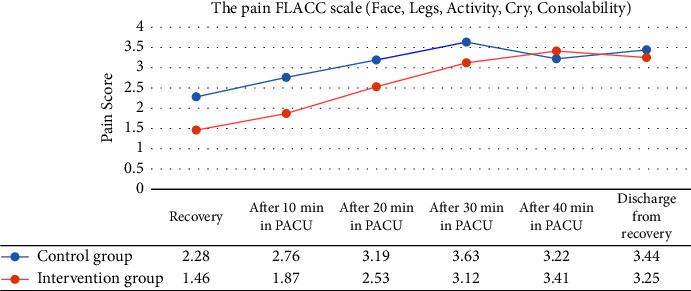
Mean pain score in both groups according to the FLACC scale. Compared with the intervention group, patients in the control group experienced high pain scores when admitted to PACU, 10, 20, 30, and 40 minutes after admission to PACU (*P* < 0.05).

Patients in the control group experienced high pain scores when admitted to PACU, 10, 20, 30, and 40 minutes after admission to PACU compared with the intervention group, and this difference was statistically significant (*P*=0.01), (*P*=0.001), (*P*=0.001), and (*P*=0.001) respectively (Figure 2). Forty minutes after PACU admission, the mean pain score in the control group was lower than the intervention, but according to the *t*-test, it was insignificant (*P*=0.064). However, when patients were discharged from the PACU, the mean pain score in the intervention group was lower than the control group (*P*=0.082). Regarding the agitation score, both groups were calm (*P*=1.00) (Table 4).

### 3.5. Antiemetic and Analgesic Consumption

**Figure 3:**
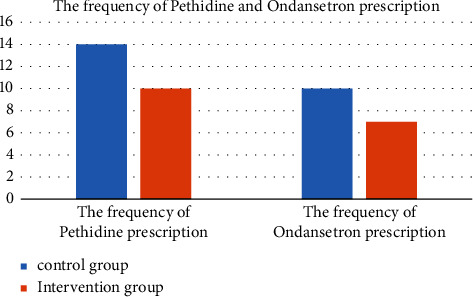
Frequency of antivomiting and analgesic consumption. The frequency of pethidine and ondansetron prescriptions in the intervention group was lower than in the control group (*P* < 0.05).

The rate of nausea and vomiting was significantly higher in the control group than in the intervention group. Hence, the control group required more ondansetron than the intervention group, which was statistically significant (*P*=0.027). Moreover, the frequency of pethidine prescription in the intervention group was significantly lower than in the control group (*P*=0.001) (Figure 3).

### 3.6. Complications

Shivering occurred in five patients in the intervention group and nine in the control groups, and it was statistically significant (*P*=0.032). Hypotension that required treatment occurred in 3 patients in the intervention group and one in the control group, which was statistically significant (*P*=0.024). Bradycardia and hypoxemia in both groups were not observed. There was no laryngospasm in both studied groups.

## 4. Discussion

Agitation following general anesthesia is usually self-limiting and often disappears immediately [8]. However, in many cases, it causes harm to oneself or others, in which case it needs treatment or even prevention [9]. Dexmedetomidine is a strong *α*2 adrenoceptor agonist that causes analgesic and sedative effects without respiratory depression [10, 11]. Therefore, dexmedetomidine can be an effective adjuvant drug in anesthesia due to the reduction of the dose of anesthetic drugs and its analgesic properties [10, 12].

In our study, dexmedetomidine effectively reduced agitation after anesthesia without prolonging the emergence from anesthesia or delaying the patients' discharge from PACU. One reason for this could be the combination of analgesic and sedative effects of this drug, which protect against agitation after anesthesia. Previous studies showed that dexmedetomidine with a dose of 0.1–0.5 *μ*g/kg effectively reduced emergence agitation after anesthesia caused by sevoflurane [16]. In this trial, we used a dose of 0.5 *μ*g/kg dexmedetomidine in bolus form after anesthesia induction which caused the frequency of emergence agitation after anesthesia in the dexmedetomidine group to be significantly lower than in the placebo group. In some similar studies, more bolus doses or even the different infusion types followed by bolus doses have been used, but the results similarly showed a significant reduction of agitation after anesthesia. In a trial conducted by Kim et al., they evaluated forty children undergoing ambulatory hernioplasty or orchiopexy; patients in the dexmedetomidine group received dexmedetomidine 1 *μ*g/kg, followed by 0.1 *μ*g/kg/h until the end of the surgery. In contrast, the saline group received volume-matched normal saline. Sevoflurane was used for induction and maintenance of anesthesia, and a caudal block was performed in all patients. Their trial demonstrated that end-tidal sevoflurane concentration in the dexmedetomidine group was significantly reduced by 23.8–67% compared to the saline group during the operation. Moreover, postoperative pain and the incidence of emergence agitation after anesthesia were lower in the dexmedetomidine group than in the saline group (5% vs. 55%, *P*=0.001) [17]. The reason for the lower prevalence of EA in the Na Young Kim study compared to our trial can be justified by considering the higher dose prescribed in the Na Young Kim study and the less painful kind of surgery or even the presence of caudal block in their study. The dose used in Kim's study and the method of injection of dexmedetomidine were different from the dose and injection method in our trial.

Ahmed Mostafa Abd El-Hamid and Hany Mahmoud Yassin [18] investigated the safety and effectiveness of intranasal dexmedetomidine (1 *μ*g/kg) in reducing the incidence and severity of emergence agitation after anesthesia in 86 patients who were scheduled for tonsillectomy or adenoidectomy under general anesthesia with sevoflurane. Results showed a significant difference in the incidence of emergence agitation after anesthesia between the intervention group and control group (6.98% and 58%, respectively, with *P*=0.001). Extubation, emergence, and discharge times were comparable in both groups. El-Sherbiny et al. [19] reported that adding dexmedetomidine to bupivacaine in sub-Tenon's block can alleviate postoperative emergence agitation and nausea and vomiting with better pain management and hemodynamic stability in pediatric strabismus surgery under sevoflurane anesthesia [19]. In KIran Sharma's trial, premedication of dexmedetomidine 10 min before induction of anesthesia at the dose of 1 *μ*g/kg in children undergoing adenotonsillectomy resulted in a favorable effect on intraoperative hemodynamics, the significant decrease in postoperative emergence agitation without causing any excessive sedation, desaturation, or any other drug-related adverse events [20]. Although the results of these investigations were somewhat similar to ours, the dose and method of administration of dexmedetomidine were utterly different. Our trial only prescribed an infusion of 0.5 *μ*g/kg of dexmedetomidine within ten minutes of induction of anesthesia. Begum et al. [21] evaluated patients aged 2–12 years undergoing lower abdominal surgeries with sevoflurane; results illustrated that both bolus and low-dose infusion of dexmedetomidine were effective for the prevention of emergence agitation after anesthesia with sevoflurane, but bolus dose of dexmedetomidine was more effective. Dexmedetomidine has numerous beneficial features as a sedative and anesthetic. The desirable physiological effects and few side effects of dexmedetomidine make it a compelling addition to anesthesia (both general and regional) for a wide range of procedures across all age groups. Kim et al. compared the preoperative administration of dexmedetomidine on the incidence and severity of emergence agitation after anesthesia in adults undergoing closed reduction of nasal bone fractures. Their study illustrated that preoperative administration of dexmedetomidine had a lower incidence of emergence agitation after anesthesia, reduced agitation severity, and a shorter duration of agitation [22]. Despite the difference in the method of dexmedetomidine administration, the type of surgery, and the age group in our study with the mentioned trial [22], the results of both studies were similar. However, the duration of agitation in our trial was not measured. The cause of emergence agitation reduction after dexmedetomidine administration is unknown, but its analgesic and sedative effects may prevent this complication. However, recent meta-analyses have shown that the analgesic effects of dexmedetomidine do not seem to play a role in this regard [23], and even in other studies, it has been stated that the low prevalence of emergence agitation after anesthesia following the administration of dexmedetomidine can be due to the lower concentrations of sevoflurane. The combination of the effects of reducing the concentration of sevoflurane due to the simultaneous administration of dexmedetomidine in the maintenance of anesthesia causes a decrease in the prevalence of emergence agitation after anesthesia [24].

Using dexmedetomidine in our trial significantly reduced antiemetic and analgesic consumption, shivering incidence, and pain intensity in the intervention group until the 40-minute postoperative time when pain scores were the same. Some studies demonstrated that pediatrics who received dexmedetomidine simultaneous with sevoflurane experienced a lower incidence of emergence agitation after anesthesia, pain, shivering, and PONV [5, 18, 25–28]. Also, they received lower amounts of antiemetic and analgesic [5, 18, 25–27]. Considering that agitation and pain evaluation in children is complicated, it should be remembered that pain may affect the level of the agitation and may be interpreted as agitation; on the contrary, pain may be considered as agitation. This issue also causes differences in presenting different frequencies of emergence agitation after anesthesia in the different studies. So we evaluated agitation at 6-time intervals by a four-point scale, and pain evaluation was performed by FLACC (faces, legs, activity, cry, and consolability). In the El-Hamid and Yassin [18] investigation, the mean FLACC pain score in the intervention group (received dexmedetomidine) was significantly lower than the control group at all six-time intervals. Also, El-Sherbiny et al. [19] revealed that the pediatric candidates for strabismus surgery who received dexmedetomidine and bupivacaine in sub-Tenon's block had lower pain scores compared to the control group (only bupivacaine).

Dexmedetomidine can cause hemodynamic variation in patients (bradycardia and hypotension are more common), and it is related to the nature of the drug (alpha2 agonist). This drug can even cause a transient increase in blood pressure, probably caused by peripheral *α*-receptor stimulation. In this study, hypotension that required intervention occurred in 3 patients in the intervention group and one in the control group, which was statistically significant (*P*=0.024). The cases of hypotension were compensated by prescribing a small amount of IV fluids. The bradycardia and hypoxemia in both groups were not observed. Hemodynamic disturbances with dexmedetomidine are common after minor surgeries, even with low doses, and reported in some clinical trials [17, 25].

## 5. Conclusion

Our trial demonstrated that the prescription of 0.5 *μ*g/kg of dexmedetomidine within ten minutes after intubation significantly reduced the emergence agitation after anesthesia, pain severity, postoperative analgesic consumption, and PONV without delaying anesthesia emerging and comparable adverse effects.

### 5.1. Study Limitations

A limitation in our trial was parental psychological consideration which was not assessed in this study, and we recommend that in the other trials, the researchers consider it.

### 5.2. Suggestions

Conducting more clinical investigations with different methods and large sample sizes in this field and combining clinical knowledge and experience to generalize the results of evidence-based studies are suggested.

## Figures and Tables

**Table 1:** Children's demographic data in both groups.

	Intervention group *N* = 38 patients	Control group *N* = 38 patients	*P* value
Gender (F/M)			
Female	12	18	0.148
Male	26	20	
Age (year)	3.97 ± 1.04	3.62 ± 1.12	0.177
BMI (body mass index) (kg/m^2^)	13.88 ± 1.39	14.55 ± 1.5	0.238
Duration of anesthesia (min)	24 ± 5.79	24.11 ± 2.51	0.431
Duration of surgery (min)	20.14 ± 2.48	18.42 ± 2.64	0.624
Emergence from anesthesia (min)	9.65 ± 5.14	7.31 ± 2.44	**0.001** ^*∗*^
Level of preoperative agitation			
1 (calm)	27 (71.05%)	30 (78.95%)	0.186
2 (not calm, but could be easily calmed)	11 (28.94%)	8 (21.05%)	
3 (moderately agitated or restless)	0 (0.0%)	0 (0.0%)	
4 (combative, excited, and disoriented)	0 (0.0%)	0 (0.0%)	

Values are presented as mean ± SD or number. There were no significant differences between demographic data in the two groups (*P* > 0.05). However, emergence from anesthesia was significantly lower in the control group (*P*=0.001)^∗^.

**Table 2:** Mean heart rate in both intervention and control groups.

Time	Intervention group	Control group	*P* value
Before injection	117.34 ± 9.57	115.97 ± 8.55	0.316
After injection	105.25 ± 13.37	78.42 ± 4.73	**0.003** ^*∗*^
5 minutes after starting the surgery	114.57 ± 11.72	76.61 ± 5.38	**0.022** ^*∗*^
10 minutes after starting the surgery	120.88 ± 3.96	119.77 ± 7.36	**0.041** ^*∗*^
15 minutes after starting the surgery	122.68 ± 5.65	120.91 ± 11.86	0.521
20 minutes after starting the surgery	122.34 ± 11.12	121.45 ± 5.90	0.334
After extubation	127.14 ± 12.47	129.38 ± 13.19	0.169
Before transferring to recovery	120.01 ± 4.47	119.62 ± 3.16	0.412
When arriving at the recovery	120.51 ± 5.78	118.94 ± 4.85	0.349
In the first 10 minutes of recovery	117.28 ± 5.75	115.97 ± 4.78	0.173
The second 10 minutes of recovery	122.16 ± 4.78	119.52 ± 3.17	0.648
The third 10 minutes of recovery	115.36 ± 6.42	117.24 ± 4.41	0.294

Values are presented as mean ± SD or number. The mean heart rate was significantly lower in the intervention group only after the injection of dexmedetomidine, 5 and 10 minutes after starting the surgery (*P* < 0.05)^∗^.

**Table 3:** Mean arterial pressure in both intervention and control groups.

Time	Intervention group	Control group	*P*-value
Before injection	68.66 ± 1.35	65.95 ± 1.52	0.415
After injection	67.42 ± 3.51	66.73 ± 2.48	0.254
5 minutes after starting the surgery	64.76 ± 1.85	70.12 ± 2.24	**0.005** ^*∗*^
10 minutes after starting the surgery	65.35 ± 2.15	69.54 ± 3.11	**0.001** ^*∗*^
15 minutes after starting the surgery	68.23 ± 2.42	70.38 ± 2.16	0.086
20 minutes after starting the surgery	70.45 ± 1.22	68.48 ± 1.67	0.171
After extubation	72.41 ± 1.39	75.22 ± 2.43	0.116
Before transferring to recovery	70.32 ± 1.79	73.42 ± 1.92	**0.021** ^*∗*^
When arriving at the recovery	68.57 ± 2.18	70.74 ± 2.53	**0.029** ^*∗*^
In the first 10 minutes of recovery	66.48 ± 1.15	70.23 ± 2.78	**0.041** ^*∗*^
The second 10 minutes of recovery	69.39 ± 2.29	68.27 ± 3.42	0.323
The third 10 minutes of recovery	72.52 ± 3.28	73.31 ± 2.65	0.418

Values are presented as mean ± SD or number. The mean arterial pressure was significantly lower in the intervention group at 5 and 10 minutes after starting the surgery, before transferring to recovery, when arriving at the recovery, and in the first 10 minutes of recovery (*P* < 0.05)^∗^.

**Table 4:** The incidence of emergence agitation in the control and intervention group.

Level of emergence agitation		Intervention group	Control group	*P* value
After extubation	1 (calm)	7 (18.42%)	5 (13.16%)	*P*=0.001
	2 (not calm, but could be easily calmed)	24 (63.16%)	22 (57.89%)	
	3 (moderately agitated or restless)	6 (15.79%)	8 (21.05%)	
	4 (combative, excited, and disoriented)	1 (2.63%)	3 (7.89%)	

Leaving the operating room	1 (calm)	11 (28.94%)	7 (18.42%)	*P*=0.001
	2 (not calm, but could be easily calmed)	20 (52.63%)	21 (55.26%)	
	3 (moderately agitated or restless)	6 (15.79%)	8 (21.05%)	
	4 (combative, excited, and disoriented)	1 (2.63%)	2 (5.26%)	

After 10 minutes in the PACU	1 (calm)	14 (36.84%)	14 (36.84%)	*P*=0.001
	2 (not calm, but could be easily calmed)	18 (47.37%)	14 (36.84%)	
	3 (moderately agitated or restless)	5 (13.16%)	8 (21.05%)	
	4 (combative, excited, and disoriented)	1 (2.63%)	2 (5.26%)	

After 20 minutes in the PACU	1 (calm)	20 (52.63%)	17 (44.74%)	*P*=0.001
	2 (not calm, but could be easily calmed)	12 (31.58%)	13 (34.21%)	
	3 (moderately agitated or restless)	6 (15.79%)	7 (18.42%)	
	4 (combative, excited, and disoriented)	0 (0.0%)	1 (2.63%)	

After 30 minutes in the PACU	1 (calm)	28 (73.68%)	25 (65.79%)	*P*=0.001
	2 (not calm, but could be easily calmed)	10 (26.31%)	11 (28.94%)	
	3 (moderately agitated or restless)	0 (0.0%)	2 (5.26%)	
	4 (combative, excited, and disoriented)	0 (0.0%)	0 (0.0%)	

After 40 minutes in the PACU	1 (calm)	38 (100.0%)	38 (100.0%)	*P*=1.00
	2 (not calm, but could be easily calmed)	0 (0.0%)	0 (0.0%)	
	3 (moderately agitated or restless)	0 (0.0%)	0 (0.0%)	
	4 (combative, excited, and disoriented)	0 (0.0%)	0 (0.0%)	

Regarding levels of emergence agitation after anesthesia, the level of agitation was significantly lower in the intervention group (*P* < 0.05), except forty minutes after staying in the recovery (*P*=1.00).

## Data Availability

All relevant data are included in the article. Additional information is available from the corresponding author on a reasonable request.
